# Autotaxin and lysophosphatidic acid_1 _receptor-mediated demyelination of dorsal root fibers by sciatic nerve injury and intrathecal lysophosphatidylcholine

**DOI:** 10.1186/1744-8069-6-78

**Published:** 2010-11-09

**Authors:** Jun Nagai, Hitoshi Uchida, Yosuke Matsushita, Ryo yano, Mutsumi Ueda, Masami Niwa, Junken Aoki, Jerold Chun, Hiroshi Ueda

**Affiliations:** 1 Division of Molecular Pharmacology and Neuroscience, Nagasaki University Graduate School of Biomedical Sciences, 1-14 Bunkyo-machi, Nagasaki 852-8521, Japan; 2 Division of Pharmacology 1, Nagasaki University Graduate School of Biomedical Sciences, 1-12-4 Sakamoto, Nagasaki 852-8523, Japan; 3 Laboratory of Molecular and Cellular Biochemistry, Graduate School of Pharmaceutical Sciences, Tohoku University, 6-3 Aobayama, Aoba-ku, Sendai 980-8578, Japan; 4 Department of Molecular Biology, The Scripps Research Institute, La Jolla, CA 92037, USA

## Abstract

**Background:**

Although neuropathic pain is frequently observed in demyelinating diseases such as Guillain-Barré syndrome and multiple sclerosis, the molecular basis for the relationship between demyelination and neuropathic pain behaviors is poorly understood. Previously, we found that lysophosphatidic acid receptor (LPA_1_) signaling initiates sciatic nerve injury-induced neuropathic pain and demyelination.

**Results:**

In the present study, we have demonstrated that sciatic nerve injury induces marked demyelination accompanied by myelin-associated glycoprotein (MAG) down-regulation and damage of Schwann cell partitioning of C-fiber-containing Remak bundles in the sciatic nerve and dorsal root, but not in the spinal nerve. Demyelination, MAG down-regulation and Remak bundle damage in the dorsal root were abolished in LPA_1 _receptor-deficient (*Lpar1*^-/-^) mice, but these alterations were not observed in sciatic nerve. However, LPA-induced demyelination in *ex vivo *experiments was observed in the sciatic nerve, spinal nerve and dorsal root, all which express LPA_1 _transcript and protein. Nerve injury-induced dorsal root demyelination was markedly attenuated in mice heterozygous for autotaxin (*atx*^+/-^), which converts lysophosphatidylcholine (LPC) to LPA. Although the addition of LPC to *ex vivo *cultures of dorsal root fibers in the presence of recombinant ATX caused potent demyelination, it had no significant effect in the absence of ATX. On the other hand, intrathecal injection of LPC caused potent dorsal root demyelination, which was markedly attenuated or abolished in *atx*^+/- ^or *Lpar1*^-/- ^mice.

**Conclusions:**

These results suggest that LPA, which is converted from LPC by ATX, activates LPA_1 _receptors and induces dorsal root demyelination following nerve injury, which causes neuropathic pain.

## Background

A significant amount of clinical evidence suggests that many demyelinating diseases are accompanied by neuropathic pain such as hyperalgesia (exaggerated pain sensations in response to mildly noxious stimuli) and allodynia (pain perception upon innocuous tactile stimuli), as seen in Guillain-Barré syndrome and multiple sclerosis[1,2]. Consistent with these clinical observations, peripheral demyelination is accompanied with thermal hyperalgesia and mechanical allodynia in *Prx *gene-deficient mice, which encodes for myelin protein in the peripheral nervous system [3]. On the other hand, there are many reports that experimental demyelination of peripheral or central neurons causes neuropathic pain behaviors [4-7].

Recently, we have reported that both neuropathic pain and demyelination are caused by sciatic nerve injury via a common pathway through LPA_1 _receptors and its downstream RhoA/Rho kinase cascade [8]. In that report, intrathecal injection of LPA was found to mimic sciatic nerve injury. This finding was supported by a recent study, in which intratrigeminal injection of LPA causes neuropathic pain-like behaviors and demyelination in rats [7]. Direct evidence for LPA-induced demyelination has been obtained in *ex vivo *cultures of primary afferent fibers [9]. Our recent study revealed that nerve injury-induced production of LPA through the action of autotaxin (ATX), which has lysophospholipase D activity and converts lysophosphatidylcholine (LPC) to LPA, is observed only in the spinal dorsal horn and dorsal root, but not in the spinal nerve, sciatic nerve and DRG for several hours [10]. Furthermore, we demonstrated that the injury-induced synthesis of LPC and subsequent conversion to LPA are both involved in the development of neuropathic pain [10,11]. However, the relationship of LPA production and demyelination following nerve injury remains to be determined. In the present study, we report that LPA_1 _receptor-mediated demyelination following nerve injury selectively occurs at the dorsal root, as seen in LPA production. In addition, we found that LPC-induced demyelination is attributed to the effects of LPA, which is converted from LPC by ATX.

## Methods

### Animals

Male mutant mice for the *lpa1 *gene (*Lpar1*^-^^/^^-^) [12], *atx*^+/- ^mice [13] and their sibling wild-type (WT) mice from the same genetic background (C57BL/6J), weighing 20-24 g, were used. They were housed at room temperature (21 ± 2°C) with free access to a standard laboratory diet and tap water. All procedures were approved by the Nagasaki University Animal Care Committee and complied with the recommendations of the International Association for the Study of Pain [14].

### Surgery and tissue preparation

Partial ligation of the sciatic nerve was performed under pentobarbital (50 mg/kg) anesthesia, following the methods of Malmberg and Basbaum [15]. Briefly, the sciatic nerve of the right limb was exposed at the high thigh of level through a small incision, and the dorsal one half of the nerve thickness at the middle part of the sciatic nerve was tightly ligated with a silk suture. Sham surgery was performed similarly, except without touching the sciatic nerve. For the electron microscopy and toluidine blue staining, three different regions of peripheral nerve, sciatic nerve and spinal nerve at 1 - 2 and 15 mm, respectively apart from the ligation site and dorsal root within 10 mm proximal to the spinal cord at the level of L4-L6 were used. For the Western blot analysis, 5 mm-long sciatic nerve including ligature site, 5 mm-long spinal nerve 13 - 18 mm apart from the ligature site and 5 mm-long dorsal root proximal to the spinal cord were isolated. Averaged wet weights of each preparation were approximately 1.0-1.5 mg.

### Drug injection

LPA (1-oleoyl-2-hydroxy-sn-3-glycerol-3-phosphate) and LPC (L-α-lysophosphatidylcholine) were purchased from Sigma-Aldrich (St. Louis, MO, USA). These drugs were dissolved in artificial cerebrospinal fluid (artificial CSF: 125 mM NaCl, 3.8 mM KCl, 1.2 mM KH_2_PO_4_, 26 mM NaHCO_3_, 10 mM glucose, pH7.4). The intrathecal injection was given into the space between spinal L5 and L6 segments according to the method of Hylden and Wilcox [16].

### Toluidine blue staining and transmission electron microscopy (TEM)

Nerve fibers were fixed with 2.5% glutaraldehyde in 0.1 M phosphate buffer (pH 7.4) overnight at 4°C. The fixed fibers were postfixed with 2% osmium tetroxide for 1 h at 25°C, dehydrated in a graded alcohol series, and embedded in Epon812. Thin sections (1 μm) were cut from each block, stained with alkaline toluidine blue, and examined by light microscopy. Ultrathin sections (80 nm thick) were cut with an Ultracut S (Leica, Wien, Austria), and then stained with uranyl acetate and lead citrate for 30 and 5 min, respectively. The stained sections were observed under an electron microscope (JEM-1200EX; JEOL, Tokyo, Japan), as previously reported [8].

### Immunohistochemical analysis

Mice were deeply anesthetized with pentobarbital (50 mg/kg), and perfused with potassium-free phosphate buffered saline (K^+^-free PBS, pH 7.4), followed by 4% paraformaldehyde solution. Nerve fibers were isolated, postfixed for 3 h, and cryoprotected overnight in 25% sucrose solution. The samples were fast-frozen in cryoembedding compound on a mixture of ethanol and dry ice and stored at -80°C until ready for use. The fibers were cut on a cryostat at a thickness of 30 μm, thaw-mounted on silane-coated glass slides, and air-dried overnight at 25°C. The sections were incubated with blocking buffer containing anti-mouse IgG (1:100; Zymed, South San Francisco, CA, USA) and 4% bovine serum albumin in PBST (0.1% Triton X-100 in K^+^-free PBS), and then reacted with anti-myelin-associated glycoprotein (MAG) antibody (1:1000; Chemicon, Temecula, CA, USA) overnight at 4°C. After washing, the sections were incubated with a secondary antibody, Alexa Fluor 488-conjugated anti-mouse IgG (1:300; Invitrogen, Eugene, Oregon, USA), for 3 h at 25°C. MAG-immunoreactivity was detected by automatic fluorescence microscopy using BZ Image Measurement software (Bio-Zero, Keyence, Tokyo, Japan).

### Western blot analysis

According to the manufacturer's instructions, non-reduced protein was used for detection of MAG, and reduced protein was used for LPA_1 _receptor and β-tubulin. Total protein (20 μg) was separated on SDS-polyacrylamide gels (12%). Primary antibodies were used at the following dilutions: mouse anti-MAG antibody (1:1000), rabbit anti-LPA_1 _receptor antibody (1:500) [17] and rabbit anti-β-tubulin polyclonal antibody (1:1000; Santa Cruz Biotechnology, Santa Cruz, CA, USA). Horseradish peroxidase-labeled anti-mouse IgG and horseradish peroxidase-labeled anti-rabbit IgG were used as a secondary antibody at a dilution of 1:1000. Immunoreactive bands were detected using an enhanced chemiluminescent substrate (SuperSignal West Pico Chemiluminescent Substrate; Pierce Chemical, Rockford, IL) for the detection of horseradish peroxidase.

### Reverse-transcription polymerase chain reaction (RT-PCR)

Total RNA was extracted from L4-6 dorsal root ganglion (DRG), dorsal root, spinal nerve and sciatic nerve using TRIzol (Invitrogen, Carlsbad, CA, USA), and 1 μg of RNA was used for cDNA synthesis. The PCR primers used in the present study were as follows: for β-actin, 5'-AGGCTCTTTTCCAGCCTTCCT-3' (forward) and 5'-GTCTTTACGGATGTCAACGTCACA-3' (reverse); for LPA_1_, 5'-ATCTTTGGCTATGTTCGCCA-3' (forward) and 5'-TTGCTGTGAACTCCAGCCA-3' (reverse). β-actin was used as an internal control. The cycling conditions for all primers were 2 min at 94°C, followed by 35 cycles of 45 sec at 94°C, 45 sec at 58°C and 70 sec at 72°C.

### Ex vivo cultures of nerve fibers

*Ex vivo *cultures of nerve fibers were performed as described previously [9]. Briefly, nerve fibers with DRG were isolated and the spinal vertebrae were carefully removed. Nerve fibers were washed with ice-cold PBS (pH7.4) containing penicillin and streptomycin, and grown in Dulbecco's modified Eagle's medium without serum. The cultures were maintained at 37°C in the presence of 5% CO_2_. LPA and LPC, used in *ex vivo *experiments, were dissolved in dimethyl sulfoxide to make a 20 mM stock solution that was stored at -80°C.

### Statistical analysis

*In vivo *experiments, the differences between multiple groups were analyzed by one-way ANOVA with Scheffe's multiple comparison post-hoc analysis. In *ex vivo *experiments, the data were analyzed using Student's *t*-test. The criterion of significance was set at *p < 0.05. All results are expressed as the mean ± SEM.

## Results

### LPA_1 _receptor-dependent demyelination of dorsal root fiber

**Figure 1:**
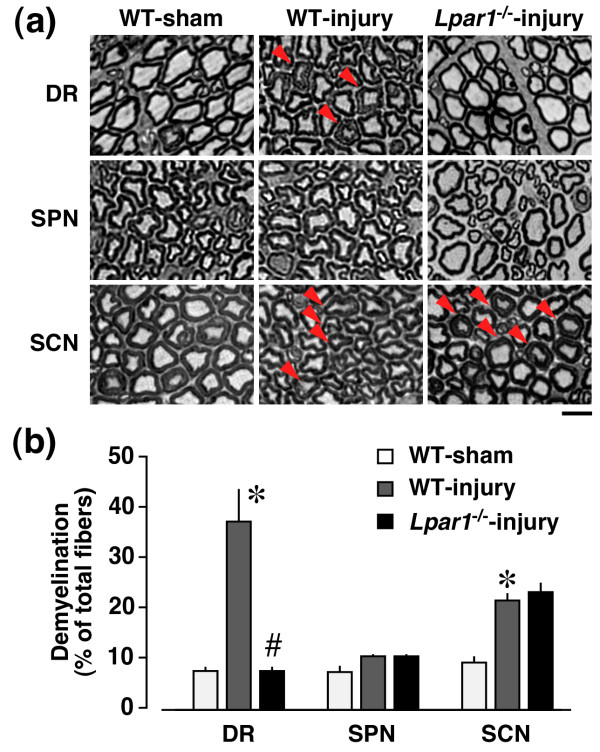
**LPA_1 _receptor-dependent and -independent demyelination after nerve injury in toluidine blue analysis**. Toluidine blue analysis was performed at day 7 post-injury. (a) Representative photographs of DR (dorsal root), SPN (spinal nerve) and SCN (sciatic nerve) regions in WT or *Lpar1*^-/- ^mice. Arrow indicates a demyelinated fiber. Scale bar, 10 μm. (b) Quantification of demyelinated fibers. Data are expressed as a percentage of total fibers in each region. *p < 0.05, *vs*. WT and sham-operated mice and #*p *< 0.05, *vs*. WT and nerve-injured mice. Results represent the mean ± SEM from 3-5 independent experiments.

To quantify injury-induced demyelination in dorsal root, spinal nerve and sciatic nerve regions, we performed toluidine blue staining, as described previously [8,9]. As shown in Figure 1, marked demyelination was observed in the dorsal root and sciatic nerve, but not spinal nerve regions at day 7 after nerve injury. Demyelination in the dorsal root and sciatic nerve was characterized by swollen myelin, decrease in myelin thickness and loss of myelin. Significant demyelination was limited within the range of 1 - 2 mm apart from the ligation site. Quantitative analysis revealed that injury increases the incidence of demyelination to approximately 35% of total fibers in the dorsal root and 20% in the sciatic nerve, but not in the spinal nerve. When *Lpar1*^-/- ^mice were used, demyelination was completely abolished in the dorsal root, but not in the sciatic nerve. However, injury did not increase the incidence of demyelination in the spinal nerve in both WT and *Lpar1*^-/- ^mice.

**Figure 2:**
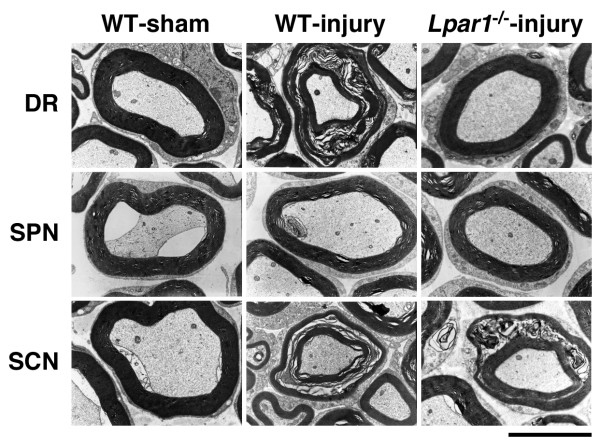
**Analysis of LPA_1 _receptor-dependent and -independent A-fiber demyelination by TEM analysis**. TEM analysis was performed at day 7 post-injury. Photographs show representative data on A-fiber myelination in dorsal root (DR), spinal nerve (SPN) and sciatic nerve (SCN) fibers in WT or *Lpar1*^-/- ^mice. Scale bar, 5 μm.

To gather more information on the morphological alterations in the myelinated fibers after injury, we performed TEM analysis. This analysis also revealed that demyelination is observed in the dorsal root and sciatic nerve, but not in the spinal nerve at day 7 after nerve injury (Figure 2). As described previously [9], the myelinated fibers were divided into two types according to the diameter of the axon and the thickness of the myelin sheath as follows: Aβ-fiber (8-15 μm in diameter and 0.6-1.0 μm thick) and Aδ-fiber (3-6 μm in diameter and 0.4-0.6 μm thick). However, there was no clear difference in the incidence of demyelination between such characterized Aβ- and Aδ-fibers. In good agreement with data obtained from toluidine blue analysis, the injury-induced demyelination was absent in the dorsal root, but not in the sciatic nerve of *Lpar1*^-/- ^mice (Figure 2). Taken together, these results strongly suggest that the LPA_1 _receptor-dependent demyelination occurs only in the dorsal root, but not spinal nerve and sciatic nerve, after nerve injury. The sciatic nerve demyelination seems to be caused by local inflammation following nerve damage [18], which is unrelated to LPA_1 _receptor-mediated neuropathic pain.

### LPA_1 _receptor-dependent damage of Remak bundles

**Figure 3:**
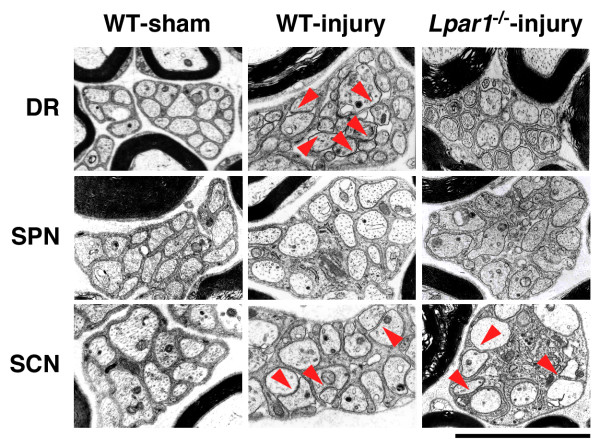
**Analysis of LPA_1 _receptor-dependent and -independent Remak bundle disruption by TEM**. TEM analysis was performed at day 7 post-injury. Photographs show representative data on C-fiber-containing Remak bundles in dorsal root (DR), spinal nerve (SPN) and sciatic nerve (SCN) fibers in WT or *Lpar1*^-/- ^mice. Arrow indicates a direct contact between C-fibers in the Remak bundle. Scale bar, 5 μm.

C-fibers without a myelin sheath bunch such as Remak bundles, and each fiber in a bundle is partitioned by Schwann cells, as described previously [9]. In the TEM analysis, we found that injury causes some damage in the partitioning of C-fibers in the Remak bundle of the dorsal root and sciatic nerve, but not in the spinal nerve at day 7 post-injury, and this damage were abolished in the dorsal root of *Lpar1*^-/- ^mice, but not in the sciatic nerve (Figure 3), being consistent with data obtained from demyelination studies (Figures 1 and 2).

### LPA_1 _receptor-dependent down-regulation of MAG protein expression

**Figure 4:**
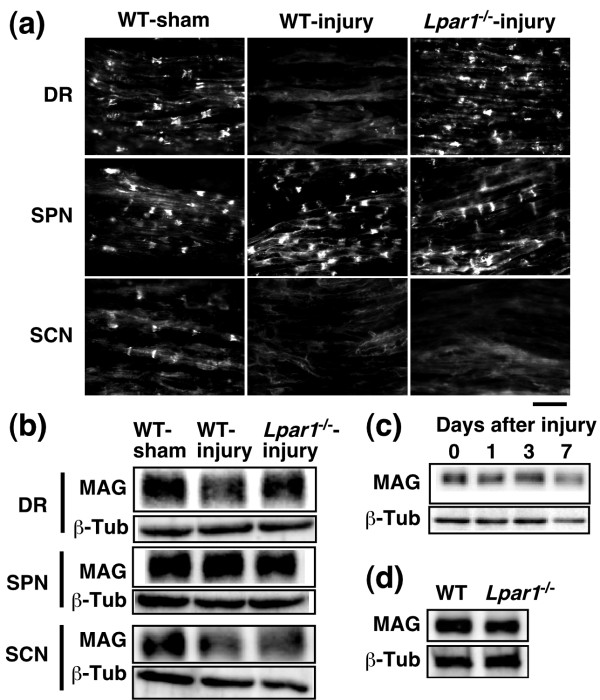
**LPA_1 _receptor-dependent and -independent down-regulation of MAG**. (a and b) The MAG expression following nerve injury in the dosal root (DR), spinal nerve (SPN) and sciatic nerve (SCN) fibers in WT or *Lpar1*^-/- ^mice at day 7 post-injury, were assessed by immunohistochemical analysis (a) and western blot analysis (b). (a) Photographs show representative data. Scale bar, 20 μm. (b) Immunoreactive signals for MAG (100 kDa) and β-tubulin (β-Tub; 55 kDa) were detected. (c) Time-course of MAG down-regulation in the dorsal root after injury. (d) Comparison of MAG expression levels between uninjured WT and *Lpar1*^-/- ^mice. Photographs show a representative image of a western blot. Data were obtained from three independent experiments.

MAG, a minor component of myelin, plays a key role in the maintenance of myelin integrity [19,20]. In the peripheral nervous system, MAG expression has been found abundantly in paranodal regions and to a lesser extent in periaxonal Schwann cell membranes [21]. Strong MAG signals were found in paranodal regions, while weaker signals were detected along axons in the dorsal root, spinal nerve and sciatic nerve regions (Figure 4a). The MAG signals were down-regulated in the dorsal root and sciatic nerve, but not in the spinal nerve, at 7 day post-injury. Moreover, we found that injury-induced MAG down-regulation is completely blocked in the dorsal root of *Lpar1*^-/- ^mice, but not in the sciatic nerve. Similar results were obtained by western blot analysis (Figure 4b). Quantitative analysis revealed that injury significantly induced down-regulation of MAG approximately 20% in the dorsal root (MAG/tubulin signals; 78.8 ± 3.8%/n = 8, compared to sham) and 30% in the sciatic nerve (67.0 ± 7.9%/n = 5), but not in the spinal nerve (109.3 ± 8.8%/n = 4). When *Lpar1*^-/- ^mice were used, down-regulation of MAG was completely abolished in the dorsal root (104.1 ± 10.3%/n = 4), but not in the sciatic nerve (62.2 ± 10.7%/n = 5). However, injury did not decrease the intensity of MAG in the spinal nerve in both WT (109.3 ± 8.8%/n = 4) and *Lpar1*^-/- ^mice (102.6 ± 6.2%/n = 4). Injury-induced MAG down-regulation in the dorsal root was slightly observed at day 1 (91.1 ± 7.1%/n = 3) and day 3 (91.8 ± 6.5%/n = 3) post-injury (Figure 4c). On the other hand, there was no difference in the basal MAG expression level in the dorsal root regions between uninjured WT (100 ± 7.3%/n = 3) and *Lpar1*^-/- ^mice (93.7 ± 5.6%/n = 3, compared to WT) (Figure 4d).

### LPA-induced ex vivo demyelination in whole regions of sensory fibers

**Figure 5:**
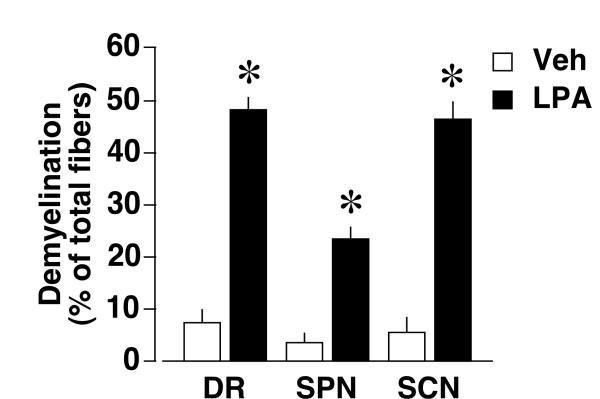
**Susceptibility of dorsal root, spinal nerve and sciatic nerve fibers to LPA-induced demyelination in the *ex vivo *culture model**. Toluidine blue analysis was performed at 24 h after the addition of LPA (1 μM) to the *ex vivo *culture of dorsal root (DR), spinal nerve (SPN) or sciatic nerve (SCN) fibers. Results are calculated as a percentage of total fibers in each region, and expressed as the mean ± SEM from 3-5 independent experiments. *p < 0.05, *vs*. vehicle (Veh)-treated groups.

To assess the responsiveness of dorsal root, spinal nerve and sciatic nerve fibers to LPA, we performed *ex vivo *cultures of nerve fibers with DRG, as described previously [9]. Toluidine blue analysis revealed that addition of LPA (1 μM) to *ex vivo *cultures causes demyelination in all regions at 24 h post-treatment, though demyelination in the spinal nerve was weaker than others (Figure 5).

**Figure 6:**
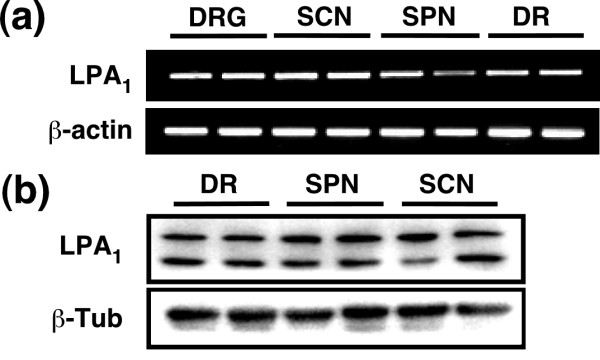
**Expression levels of LPA_1 _receptor in the dorsal root, spinal nerve and sciatic nerve fibers**. (a) The mRNA expression levels of LPA_1 _receptors in the dorsal root ganglion (DRG), dorsal root (DR), spinal nerve (SPN) or sciatic nerve (SCN) fibers *in vivo*, assessed by RT-PCR. β-actin was used as an internal control. Photographs show the representative image of RT-PCR. Data were obtained from three independent experiments. (b) Protein expression levels of LPA_1 _receptors in the dorsal root (DR), spinal nerve (SPN) or sciatic nerve (SCN) fibers *in vivo*, using western blot analysis. β-tubulin (β-Tub) was used as an internal control. Photographs show the representative image of western blot. Data were obtained from three independent experiments.

Previously, we have reported that the LPA_1 _receptor-mediated Rho-Rho kinase pathway is crucial for LPA-induced demyelination [8]. Using RT-PCR analysis, however, we found that the LPA_1 _receptor is ubiquitously expressed in the dorsal root, spinal nerve, sciatic nerve and DRG (Figure 6a). Western blot analysis also showed similar expression patterns of the LPA_1 _receptor (Figure 6b).

### ATX-mediated demyelination following injury

**Figure 7:**
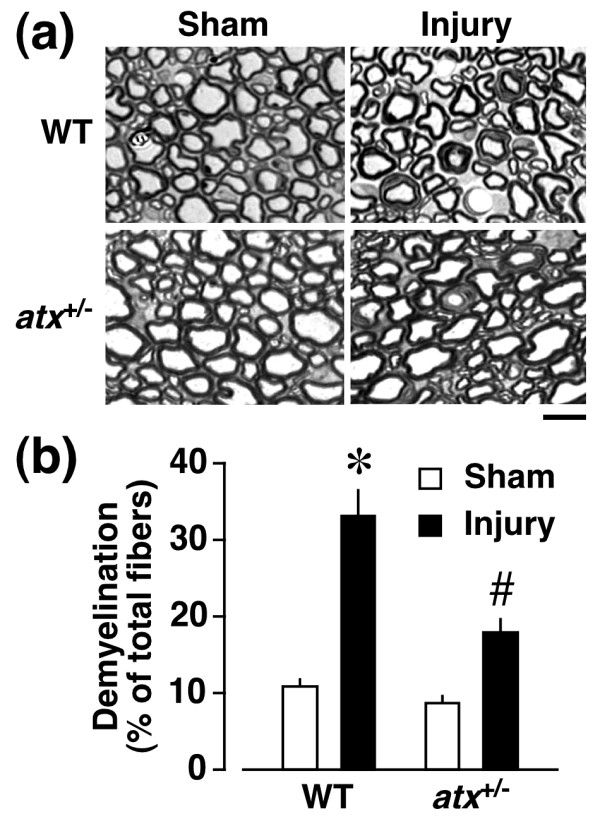
**Attenuation of injury-induced dorsal root demyelination in *atx*^+/- ^mice**. Toluidine blue analysis was performed at day 7 post-injury in WT or *atx*^+/- ^mice. (a) Photographs show representative data in the dorsal root. Scale bar, 10 μm. (b) Quantification of demyelinated fibers. Results are calculated as a percentage of total fibers, and expressed as the mean ± SEM from 3-5 independent experiments. *p < 0.05, *vs*. WT and sham-operated mice and #*p *< 0.05, *vs*. WT and nerve-injured mice.

As ATX plays a key role in nerve injury-induced LPA production and neuropathic pain [10,11], we used *atx*^+/- ^mice to examine the involvement of *de novo *synthesis of LPA in dorsal root demyelination. In the toluidine blue analysis, the injury-induced demyelination level in *atx*^+/- ^mice was reduced to approximately half in WT mice (Figure 7), being consistent with the change of LPA production and neuropathic pain in *atx*^+/- ^mice [10,11]. There was no significant difference in the morphology of myelinated fibers and basal incidence of demyelinated fibers between *atx*^+/- ^and WT mice.

### LPC-induced ex vivo demyelination in the presence of ATX

**Figure 8:**
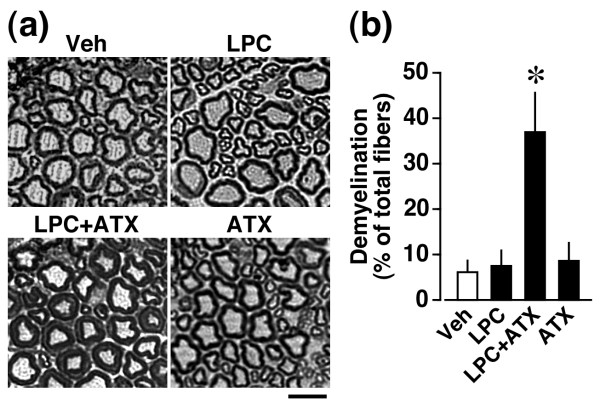
**LPC-induced dorsal root demyelination mediated through ATX**. Toluidine blue analysis was performed at 24 h after the addition of 100 ng/ml of LPC to *ex vivo *cultures of DR fibers in the presence or absence of ATX (30 ng/ml). (a) Photographs show representative data. Scale bar, 10 μm. (b) Quantification of demyelinated fibers. Results are calculated as a percentage of total fibers, and expressed as the mean ± SEM from 3-5 independent experiments. *p < 0.05, *vs*. vehicle (Veh)-treated group.

Based on previous observations that LPC-induced neuropathic pain behaviors are abolished or markedly attenuated in *Lpar1*^-/- ^or *atx*^+/- ^mice, respectively [22], we examined the effects of LPC in the presence and absence of recombinant ATX on demyelination in *ex vivo *experiments. As shown in Figure 8, toluidine blue analysis revealed that LPC at a concentration as low as 100 ng/ml (approximately 0.2 μM) in the presence of ATX (30 ng/ml) markedly increases the incidence of demyelination of dorsal roots to 35% of total myelinated fibers, being equivalent to the level of *ex vivo *dorsal root demyelination by LPA (1 μM) treatment and *in vivo *dorsal root demyelination following nerve injury. There was no significant change in the extent of demyelination by LPC or ATX alone.

### ATX- and LPA_1 _receptor-mediated demyelination by LPC

**Figure 9:**
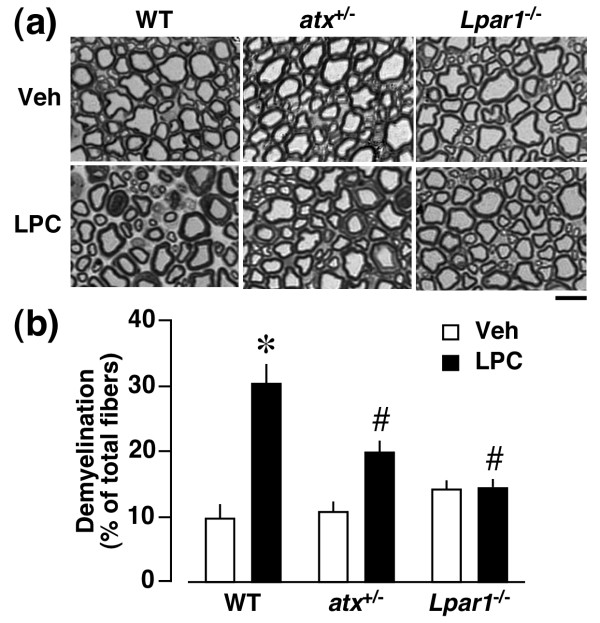
**LPC-induced dorsal root demyelination mediated through ATX and LPA_1 _receptors *in vivo***. Toluidine blue analysis was performed at day 4 after the intrathecal injection of LPC (15 μg) in WT, *atx*^+/- ^or *Lpar1*^-/- ^mice. (a) Photographs show representative data in the dorsal root. Scale bar, 10 μm. (b) Quantification of demyelinated fibers. Results are calculated as a percentage of total fibers, and expressed as the mean ± SEM from 3-5 independent experiments. *p < 0.05, *vs*. WT and vehicle (Veh)-treated mice and #*p *< 0.05, *vs*. WT and LPC-treated mice.

When LPC (15 μg, approximately 30 nmol) was given intrathecally, there was an increase in dorsal root demyelination at day 4 post-injection. The level was approximately 30% (Figure 9), being equivalent to that (30%) by intrathecally administered 1 nmol of LPA [8]. The demyelination was significantly attenuated in *atx*^+/- ^mice, and abolished in *Lpar1*^-/- ^mice (Figure 9).

## Discussion

Myelination is known to insulate nerve fibers for rapid nerve conduction. Demyelination is thought to reduce conduction velocity, thereby causing sensory and motor dysfunction in peripheral demyelinating diseases, including Charcot-Marie-Tooth disease [23]. However, there are reports that demyelination leads to neuronal hyperexcitability associated with neuropathic hyperalgesia and allodynia [3,4]. Thus, some secondary events after demyelination may be included in this functional difference *in vivo*. In agreement with these findings, the formation of electrical crosstalk (emphatic interaction) or sprouting after demyelination has been implicated in neuronal hyperexcitability [24-26].

In the present study, we found that nerve injury induces demyelination and damages Remak bundles in the dorsal root. These changes were abolished in *Lpar1*^-/- ^mice, suggesting that LPA causes these changes via Schwann cells. Indeed, we have reported that the addition of LPA causes demyelination and damage of Remak bundles in dorsal root fibers in *ex vivo *culture experiments [9]. Regarding the molecular basis for demyelination, we have proposed that LPA down-regulates gene and protein expression of compact myelin proteins, such as myelin basic protein, peripheral myelin protein 22 and myelin protein zero [8,9]. Here, we further observed down-regulation of MAG, which inhibits nerve sprouting through regulation of actin assembly by the Rho-Rho kinase-Lim kinase pathway [24]. Indeed, MAG down-regulation following nerve injury is reported to induce axonal sprouting [27]. This action seems to be related to the challenging proposal that the synaptic reorganization involved in neuropathic allodynia may be mediated by axonal sprouting in the DH [24,28].

In the present study, we demonstrated that the sciatic nerve injury caused LPA_1 _receptor-mediated demyelination and its underlying MAG down-regulation only in the dorsal root, but not spinal nerve or sciatic nerve (Figures 1, 2, 4). In the sciatic nerve, there was LPA_1_-independent demyelination. However, as LPA-mediated *in vitro *(*ex vivo*) demyelination was observed all in dorsal root, spinal nerve and sciatic nerve (Figure 5), it seems that machineries required for LPA-mediated demyelination are present in these three regions. Therefore, the dorsal root-specific LPA_1_-mediated demyelination would be explained by the speculation that LPA is produced in spinal cord and transferred to the dorsal root, where demyelination occurs. However, there is still a possibility that dorsal root demyelination is attributed to the action of LPA derived from dorsal root. Regarding the proposal that LPA involved in the dorsal root demyelination comes from the spinal dorsal horn, we have reported that the intense stimulation of spinal dorsal horn *in vitro *causes *de novo *production of LPA [29].

On the other hand, there are reports that demyelination at the nerve-injured site, is caused by an activation of matrix metalloproteinase-9 [30,31]. However, this activation is unlikely related to the action of LPA, since no significant LPA production in the sciatic nerve was observed by nerve injury [10]. Furthermore, metalloproteinase inhibition did not affect MAG down-regulation in the dorsal root following intrathecally administered LPA [32], which mimics the nerve injury in terms of induction of neuropathic pain and its underlying mechanisms [8].

The present study demonstrated that sciatic nerve injury did not cause demyelination in the spinal nerve. The partial ligation of sciatic nerve was performed within 1 mm-long fiber portion in sciatic nerve, and significant demyelination was limited within the range of 1 - 2 mm apart from the ligation site. As isolated spinal nerve region is over 10 mm apart from the ligation site of the sciatic nerve, it is unlikely that inflammatory effects in sciatic nerve, if any, affect on the spinal nerve region.

Accumulating evidence has shown that LPC, a precursor of LPA, induces focal demyelination, thereby causing neuropathic pain conditions [4,33]. These studies topically applied as much as 1-2% (10-20 μg/μl) of LPC to the nerve fibers, and this dose is reported to be toxic to myelinating cells due to the detergent-like properties of LPC [34]. On the other hand, in the present study we observed that intrathecal administration of 0.3% (3 μg/μl, 5 μl) LPC, which in turn would be diluted by circulating CSF, causes dorsal root demyelination. In addition, LPC-induced demyelination was attenuated to approximately 50% in *atx*^+/- ^mice and abolished in *Lpar1*^-/- ^mice. Similar attenuation was observed in injury-induced demyelination in *atx*^+/- ^mice, being consistent with the finding that injury-induced neuropathic pain and LPA production in the dorsal root are also attenuated to approximately 50% in *atx*^+/- ^mice [10]. In the present study, we failed to detect any significant demyelination of nerve fibers in the *ex vivo *study with LPC, but further addition of recombinant ATX showed significant demyelination at equivalent levels to the case with LPA. The reason LPC did not cause demyelination, may be attributed to the loss of ATX throughout the isolation and preparation processes of dorsal root fibers in the *ex vivo *study, consistent with LPA production [29,35]. However, we have demonstrated that significant amounts of ATX (5 μg/mL) exist in CSF *in vivo *[29]. Thus, we have successfully provided evidence that demyelination occurs through the conversion of LPC into LPA by ATX *in vivo*.

Lastly, although the relationship between LPA- or injury-induced demyelination and development of neuropathic pain remains to be fully elucidated, we have recently observed that calpain inhibition abolishes down-regulation of MAG in the dorsal root and neuropathic pain after intrathecal administration of LPA or nerve injury [32]. However, further studies will clarify the role of demyelination underlying injury-induced neuropathic pain.

## Conclusions

The present study demonstrated that LPA_1 _receptor signaling following injury causes demyelination specifically at the dorsal root, which is consistent with *de novo *LPA production. In addition, we found that demyelination by topical application of LPC is attributed to the action of LPA after conversion by ATX.

## List of abbreviations

ATX: autotaxin; *atx*^*+/-*^: mice heterozygous for autotaxin; CSF: cerebrospinal fluid; DH: dorsal horn; DRG: dorsal root ganglion; GAPDH: glyceraldehyde-3-phosphate dehydrogenase; i.t.: intrathecal; LPA: lysophosphatidic acid; *Lpar1*^*-/-*^: LPA_1 _receptor-deficient mice; LPC: lysophosphatidylcholine; MAG: myelin-associated glycoprotein; PBS: phosphate buffered saline; RT-PCR: reverse-transcription polymerase chain reaction; TEM: transmission electron microscopy; WT: wild-type

## Competing interests

The authors declare that they have no competing interests.

## Authors' contributions

JN contributed to the experiment on demyelination and the writing the manuscripts. H Uchida participated in the RT-PCR study and writing the manuscripts. YM performed experiments on demyelination. RY performed experiments on immunohistochemistry. MU and MN participated in the writing the manuscripts. JC generated *Lpar1*^*-/- *^mice. JA generated recombinant ATX and *atx*^*+/- *^mice. H Ueda is responsible for the experimental design and writing the manuscript. All authors read and approved the final manuscript.
